# Meta-Analysis of Fluid Intelligence Tests of Children from the Chinese Mainland with Learning Difficulties

**DOI:** 10.1371/journal.pone.0078311

**Published:** 2013-11-13

**Authors:** Fang Tong, Tong Fu

**Affiliations:** 1 Capital Institute of Pediatrics, Beijing, P. R. China; 2 Beijing Information Science and Technology University, Beijing, P. R. China; Fondazione IRCCS Ca' Granda Ospedale Maggiore Policlinico, Università degli Studi di Milano, Italy

## Abstract

**Objective:**

To evaluate the differences in fluid intelligence tests between normal children and children with learning difficulties in China.

**Method:**

PubMed, MD Consult, and other Chinese Journal Database were searched from their establishment to November 2012. After finding comparative studies of Raven measurements of normal children and children with learning difficulties, full Intelligent Quotation (FIQ) values and the original values of the sub-measurement were extracted. The corresponding effect model was selected based on the results of heterogeneity and parallel sub-group analysis was performed.

**Results:**

Twelve documents were included in the meta-analysis, and the studies were all performed in mainland of China. Among these, two studies were performed at child health clinics, the other ten sites were schools and control children were schoolmates or classmates. FIQ was evaluated using a random effects model. WMD was −13.18 (95% CI: −16.50–−9.85). Children with learning difficulties showed significantly lower FIQ scores than controls (*P*<0.00001); Type of learning difficulty and gender differences were evaluated using a fixed-effects model (I^2^ = 0%). The sites and purposes of the studies evaluated here were taken into account, but the reasons of heterogeneity could not be eliminated; The sum IQ of all the subgroups showed considerable heterogeneity (I^2^ = 76.5%). The sub-measurement score of document A showed moderate heterogeneity among all documents, and AB, B, and E showed considerable heterogeneity, which was used in a random effect model. Individuals with learning difficulties showed heterogeneity as well. There was a moderate delay in the first three items (−0.5 to −0.9), and a much more pronounced delay in the latter three items (−1.4 to −1.6).

**Conclusion:**

In the Chinese mainland, the level of fluid intelligence of children with learning difficulties was lower than that of normal children. Delayed development in sub-items of C, D, and E was more obvious.

## Introduction

The advancement of children's mental development is drawing more and more attention. The incidence of syndromes involving learning difficulties among Chinese children ranges from 5–16%, and the established literature relies primarily upon the results of intelligence tests

Cartel divided general intelligence into two categories, fluid intelligence and crystallized intelligence. Crystallized intelligence is the intellect gained through mastery of social and cultural experiences, such as lexical concepts, speech comprehension, common sense, and other information stored as memory. This intelligence remains relatively stable throughout a person's life. However, Wechsler measuring was listed by Rushton as a further sub-measurements of crystallized intelligence. There are neural connections between fluid and crystallized intelligence [1], [2]. Fluid intelligence involves an innate ability to conduct activities requiring intelligence, such as learning and problem-solving, which rely on innate endowment and improve as the nervous system matures. It is a basic human capability, influenced by genetic factors. It is characterized by the ability to comprehend unfamiliar things, to react quickly, and to accurately assess relationships between concepts. Many experimental measurements have seemed to show racial differences between children's fluid intelligence development. Asian score 5–9 points higher than Caucasians on the British norm 100. Mexican Mestizos tend to score 94.3 points out of 100, and Mexicans of Mexican Indian ancestry aged 7–10 scored 83.3 points [3], [4], [5]. During the pre-school period, gender differences in fluid intelligence do not tend to be visible, but when children pass age 7, boys gradually tend to outscore girls by about 4–6 points, and this difference persists into adulthood [4], [6]. A positive correlation was observed with working memory [3]. It is affected by education and culture in a negligible way. The development of fluid intelligence has been shown to be closely related to age. For most people, the development of fluid intelligence for most people peaks between the ages of 20 and 30, after which it decreases [7]. Raven measurements are considered to be a suitable means of measuring genetic fluid intelligence.

The Raven used in Chinese mainland was based on the British psychologist J.C. Raven's combined type of non-verbal intelligence test compiled in 1938. This is used to deal with fluid IQ in China [8], [9]. The test itself was developed in 1989 and 1997 by Dan Li and Dong Wang of East China Normal University based on a previous version by Hou-can Zhang of Beijing Normal University, published in October of 1985 [10]. These checks are time- and labor-intensive. Usually, the samples are small and only weakly representative. Due to the different levels of control and different testing conditions and sometimes the different purposes and geographical locations of the studies, not only children with learning difficulties but also children in the normal comparative group are likely to differ across studies. It is therefore necessary to integrate all reports as comprehensively as possible.

## Methods

### Protocol and registration

Based on a small sample of multi-faceted examinations of children with learning difficulties screened out from the original clinical, a pre-established program was implemented and data were entered into an Excel database in pre-pressed format. Offline analysis was performed with free software **ReverMen 4.2.2.**

### Documents inclusion criteria


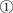
 The study participants were school-age children (6–16 years old). 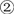 They were of Han ethnicity. 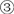 The study was comparative study, evaluating both normal children and children with learning difficulties. 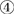 The general fluid intelligence tests were performed with the Combined Raven's Test (CRT). 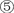 Requests for the original information involved publicly and privately reported general fluid intelligence testing of children with learning difficulties. Total (**FIQ**) scores and results of sub-measurements were reported. 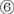 Languages were limited to Chinese and English., 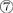 All studies were performed in mainland China. 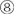 Repeatedly published documents were included among recently published documents.

### Document exclusion criteria


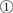
 The published article was written in dialect, not standard language. 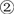 Either the control group contained non-normal children served as controls or comparisons were only made among groups of children with learning difficulties, even if those difficulties were of different levels of severity. 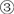 A repeat publication. 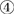 Brain functions delay were evaluated in the study.

### Diagnostic criteria


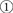
 International Classification of Diseases, 10^th^ Edition, ICD-10. 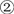 Chinese Criteria for Mental Diseases, 3^th^, CCMD-3. 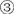 The Pupil Rating Scale, PRS, the total score is <65 points and the scaled verbal score is <20 points. 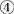 Through CRT and other intellectual screening tests, IQ> = 70–85. 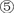 Sensory impairments, neuropsychiatric disorders, and somatic diseases, including attention deficit hyperactivity disorder and organic brain disease, were excluded. 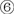 Teacher rating: the average score of core subjects (Chinese, mathematics, English) below 10% of the class score, or head teacher rating: having learning disabilities for more than 1 year, or parents rating: being unable to finish homework independently. The main diagnostic criteria were items 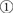,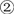, and 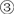, Basic diagnosis was based on items 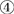
–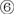 and items 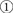, 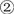, and 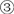 were unmentioned.

### CRT basic contents

FIQ is the total deviation IQ. It is made up of six segments: A (perception identifying ability), B (graphical comparison, resemblance comparing capacity), C (graphics combining, comparing reasoning ability), D (graphics integration, series correlating capacity), and E (graphics interchange, abstract reasoning ability). There were 12 questions per segment. Zhang HC (Beijing Normal University, Revision of October 1985) published 5 sub-measuring items with 60 questions (excluding AB); Li D. and Wang D. (East China Normal University, 1989, Tianjin Normal University, Second Revision of 1997) published 6 sub-measuring items with 72 questions.

### Sources of information and search process

PubMed, MDConsult, the China National Publication Linker (cnpLINKer), the Chinese Knowledge Resource Integrated Database, and the Wan Fang Biomedical Journals Database were searched using the following Chinese and English keywords: “learning difficulty,” “children,” and “CRT” within the time from establishment of the database to November 2012. The download addresses are as follows: http://www.mdconsult.com; http://www.yz365.com/Pubmed; http://192.168.106.13/kns50/; http://g.wanfangdata.com.cn

### Study selection

Hundreds of studies were screened and inappropriate documents were filtered out using exclusion criteria. Studies that met the inclusion criteria were accessed. Two authors developed indicators and tables. After displaying the indicators of each study, data were transcribed in Microsoft Excel, copied into **RevMan**, and used to produce flow charts.

### Content extracted from data

The first author, publication date, author's organization, sample size, Chinese version of CRT, diagnostic criteria, form of publication, FIQ value, sub-item scores, and matching conditions and the subjects' geographical location, age, and learning difficulties were recorded.

### Quality assessment criteria of documents

The Newcastle Ottawa scale (NOS), a tool for document evaluation of non-randomized comparing study, was used to evaluate the quality of the included documents: 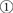 The method of selection of the case group and control group, including case definitions, representation, and diagnosis, the definition and selection of the comparative group. 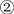 Comparability between case group and comparative group. 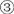 Methods of data assessment: investigations and assessment methods; whether the methods of investigating of cases and controls group were the same; data on response rate. There was a total of eight NOS evaluation criteria that scored 10 points. Documents that scored 8 points or more were considered high quality, documents that scored 7 points were considered higher-quality documents, documents that scored 6 points were considered medium quality, and documents that scored under 5 points were considered low quality.

### Statistical methods

The degree of publication bias was assessed with a funnel diagram. Synthetic intelligence scores were measured and data are displayed with weighted mean difference (WMD) and 95% CI. Statistical heterogeneity analysis was performed on the studies evaluated here. When *P*≤0.1 there was significant heterogeneity between studies. Quantitative analysis was performed by adopting I^2^ toward heterogeneity; when I^2^≤25%. The heterogeneity between study results was lower. When I^2^ was between 26 and 50%, there was moderate heterogeneity; when I^2^>50%, there was a high degree of heterogeneity. When there was no heterogeneity between the results, a fixed effects model was used. In other cases, a random effects model was used to describe the results. RevMan 4.2.2 was used for meta-analysis. *P*<0.05 indicated that the difference was statistically significant.

### Bias between studies

Children with learning difficulties experience heterogeneous syndromes. Measurement strategies that are not double-blind or did not take random error or geographical differences into account can all introduce bias. I^2^ grouping analysis can be used to evaluate the results.

### Supplementary analysis (subgroup analysis)

A meta-analysis of the scores reported in six sub-measurement items (**A**, **AB**, **B**, **C**, **D**, **E**) was performed.

## Results

### General conditions

**Figure 1:**
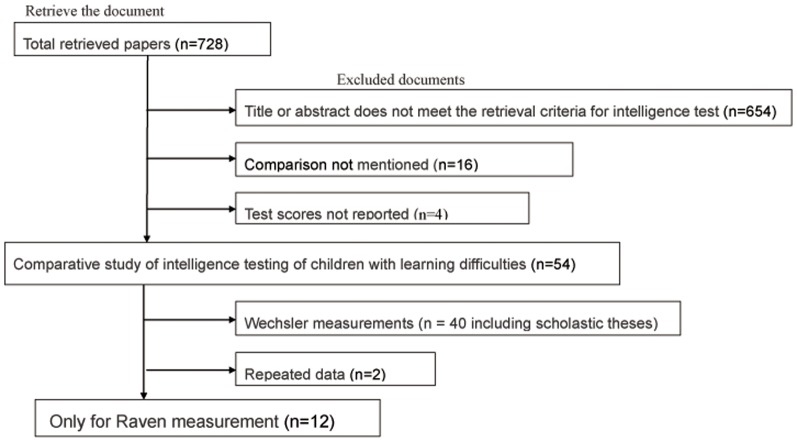
Flowchart of document retrieval.

Initially, 728 documents were retrieved, and 12 of them had been published in Chinese [11]–[22]. These 12 were entered into the meta-analysis (Figure 1). The basic characteristics of the documents are shown in Table 1: Four documents were taken from *Chinese Mental Health*, two from *China School Health*, and the others from the *Chinese Journal of Behavioral Medical Science*, *Journal of Health Psychology*, *Chinese Eugenics and Genetic Magazine*, *Chinese Journal of Child Health Care*, *Chinese Journal of Endemiology*, and *Practical Preventive Medicine*. Ten of these studies took place at schools [11]–[17], [20]–[22], two in clinics [18], [19]. The control groups were normal children from the same classes and grades, and always from the same school. They were always of the same age and gender. There was a total of 1,098 children with learning difficulties and 2,008 controls. The male/female ratio was approximately 3:1. All survey areas were in eastern and southern China (Table 1).

**Table 1:** Basic characteristics of the twelve studies evaluated here.

Author	Area	Version of Raven	Diagnostic regulations	Site	Number of cases	Male	Female	Grade	Average age	Control criteria	Number of cases	Male	Female	Age
Jing Jin1996	Guangzhou	CRT (South China Normal University 1989)	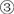 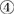 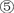 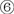	2 primary schools	79	55	24	grade 4–6	11.81±1.11	Matching by grade, age, and gender	79	55	24	same age same gender
Fang Yaohua 1998	Guangzhou	CRT (South China Normal University 1989)	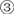	Primary school of Guangzhou City primary school	76			grade 4–6		Compare with above Jing Jin	79	55	24	4–6 grade
Yang Zizhen 2000	Shanghai	CRT (South China Normal University 1989)	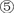 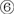	Some primary school	50			grade 4–5	9-4∼10-9	Matching with a 1:1 ration for age and gender within one school	50			same age same gender
Xu Shaojun 2005	Hefei	CRT (1989)	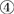 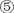 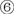	Some primary school in Hefei	55	36	19	grade 2–6		Matching with a 1:1 ratio for gender within one class	55	36	19	same age same gender
Li Juan 2006	Wuhan	WD Wangdong, 1997 CRT (Tianjin Medical University 1997)	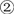 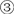 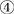 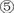 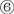	Screening some primary school	90	60	30	grade 1–6		Matching at a 1:1 ratio within grade	90	60	30	Similar in age and gender
Liang Junlin 2000	Lechang City	CRT (South China Normal University 1989)	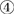 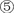 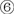	Screening at 4 middle schools	119	85	34		14.13±1.09	Same class	115	42	73	13.52±1.06
Cai Zhengyi 2000	Shanghai	CRT (South China Normal University 1989)	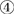 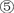	2 ordinary primary schools	130	Non	Non	grade1–5		Screening the remaining children	1180	Non	Non	Same age
Sun Chaoqi 2000	Suzhou	CRT (South China Normal University 1989)	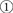	Outpatient service at this hospital	58	Non	Non		8–10 years old	Random selection	42	Non	Non	Similar in age and gender
Zhang Fangrong 2000	Shenzhen	Combined Raven Test	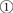 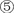 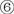	Outpatient service at this hospital	93	67	26		8.7±1.7	Randomly match according to student ID No. in some primary school	93	63	30	Same age
Liang Xiaohong 2006	Guangzhou	CRT (South China Normal University 1989)	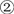	Some primary school	58	30	28	grade 3–5	9–11 years old	Matching in the same class	58	30	28	Same class
Wang Zhong 2010	Zhanjiang, Guangdong	CRT (South China normal university –Li Dan)	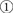	3 primary schools in Zhanjiang	286	225	61	grade 1–6	Grouping according to grade	Matching with the ratio of 1:2∼1 according to grade, gender and age	171	129	42	Same class
Wang Engguo2008	Nanjing	-Raven Standard Reasoning manual-Zhang Houcan Beijing Normal University 1985	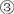 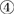 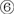	4 middle schools in Nanjing	82	46	36	second year of middle school		Same grade	28	16	12	Same grade

### Bias within each study

**Figure 2:**
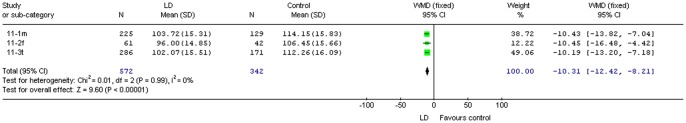
Gender differences.

**Figure 3:**
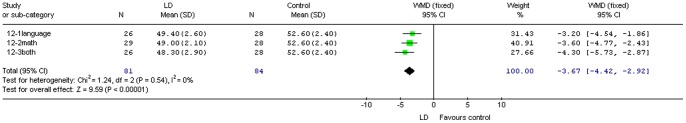
Differences between different types of learning difficulties.

No. 11 study [21] reported based on different gender and made comparison between boys, girls and overall situations, and no significant difference between gender was found in research (Figure 2); No. 12 study [22] reported by language difficulties, math difficulties and mixed difficulties, and no significant difference existed among the raw scores of different types of learning difficulties (Figure 3) (All I^2^ are 0)

### Document quality assessment

Two studies were about screening [12], [20]. Another two had diagnosis with insufficient reference [13], [17]. The hospital study site didn't describe whether the case was consecutive or not. One study did not involve screening [17]. The definition and selection processes included in the comparison group were appropriate. Ten studies were performed by schools, two groups were not comparable in either age or grade or other factors, and the CRT of these two groups involved the same scaling test, but the response rates were not described and the methods of description were blind. There were four studies with **NOS** scores above 8 points [11], [14], [15], [16], one study with 7 points [12], four studies with 6 points [17], [19], [21], [22], and two studies with 5 points or fewer [18], [20].

### Publication bias

**Figure 4:**
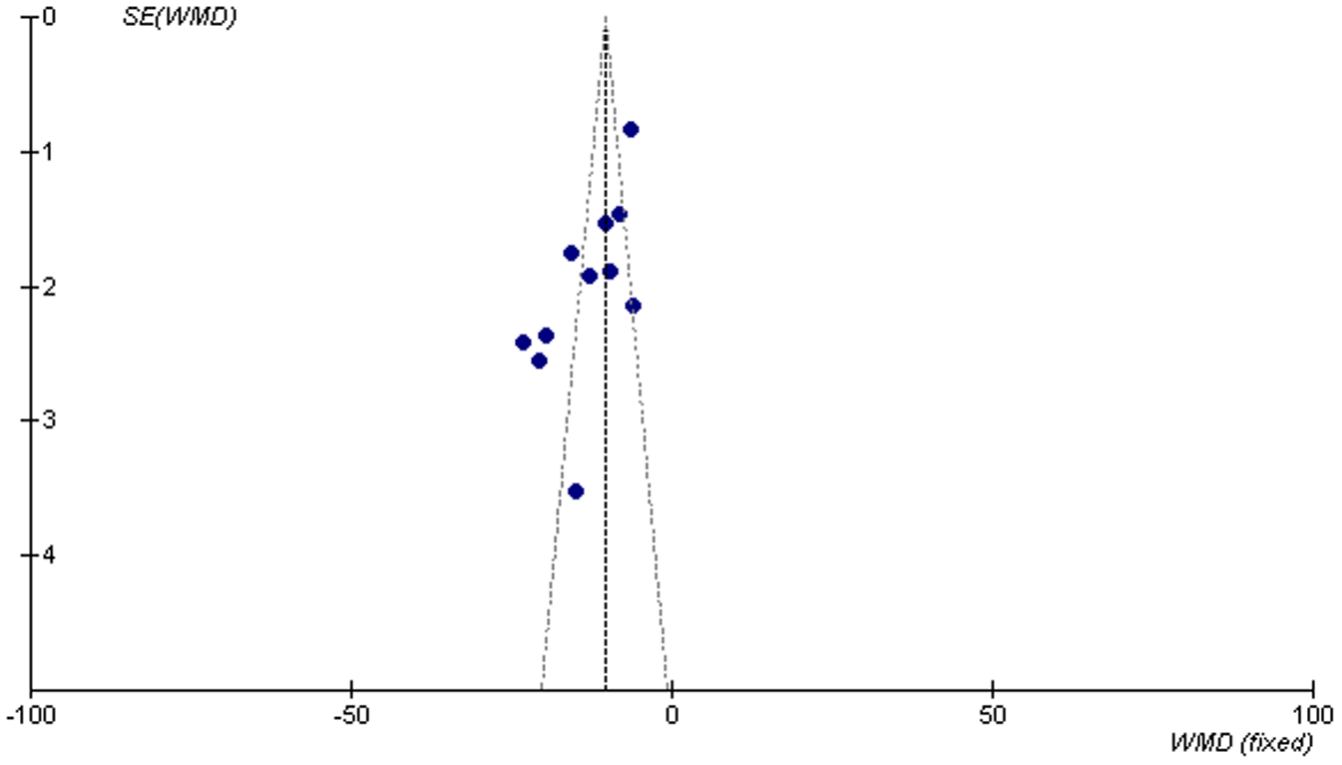
FIQ funnel diagram.

Figure 4 is based on of the eleven reported studies on FIQ. The graphic is asymmetrical, indicating possible publication bias.

### Results of FIQ meta-analysis

**Figure 5:**
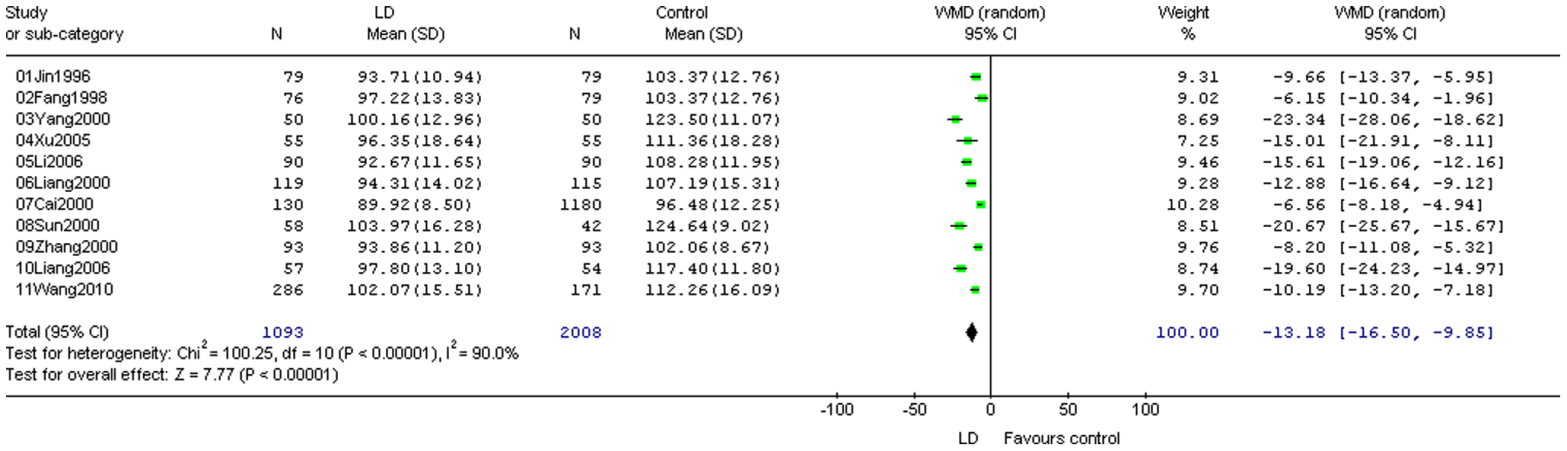
Meta-analysis of the comparison between learning difficulties group and comparative groups.

One study did not report FIQ [22]. Through integrated quantitative analysis of the results of other eleven studies, the heterogeneity test I^2^ was found to be 90.0%, and there were significant statistical heterogeneity between studies, and random effects model was used to merge the results. Meta-analysis showed **WMD** = −13.18 (95% CI −16.50–−9.85) (Figure 5), and groups with learning difficulties showed significantly lower results than the control group (*P*<0.00001).

### Analysis of heterogeneity causes

Because FIQ heterogeneity was found to be highly significant among the documents evaluated here, it may be associated with the following factors: 1) Different diagnostic criteria. Four of the documents involved primary diagnostic indicators [11], [18], [19], [21], five met the basic diagnostic criteria [13], [14], [15], [16], [22], and two adopted screening criteria [12], [20]. 2) Different research purposes. Four studies focused on research into general intelligence in individuals with learning difficulties [11], [12], [13], [21], two were about major related behaviors [14], [18], three were about related comprehensive factors [15], [16], [19], and the other three were related to personality [17], iodine exposure [20], and the processing of different types of information [22]. 3) Different research scenes. Ten studies were performed at schools, two were performed in hospitals. The matching conditions of comparative groups at schools were significantly better than at hospitals. 4) Different **CRT** versions. Ten studies adopted East China Normal University 1989 version, one adopted the 1997 reprint version of Tianjin University [19]. 5) Moderate heterogeneity in the two studies that evaluated conduct (I^2^ = 41.0%), all others I^2^>50% and *P*<0.0001, suggesting significant heterogeneity among the documents (Table 2). Meta-analysis showed significant differences; individuals with FIQ learning difficulties showed significantly lower scores than the control group.

**Table 2:** Grouping analysis of FIQ document heterogeneity.

Grouping	Research code	heterogeneity		Weighted value difference (95%CI)	*P*
		*P*	I^2^ (%)		
Sources:					
School (random)	[1]–[7], [10], [11]	<0.00001	90.2	−13.00 (−16.72, −9.27)	<0.00001
Outpatient services(random)	[8], [9]	<0.00001	94.4	−14.26 (−26.43, −2.05)	0.02
Raven's testing edition:					
only 1989 (random)	[1], [2], [4], [6]–[11]	<0.00001	86.4	−11.71 (−14.83, −8.59)	<0.00001
Type of learning:					
difficulties (fixed)	[12]	0.54	0	−3.67 (−4.42, −2.92)	<0.00001
Gender (fixed)	[11]	0.99	0	−10.31 (−12.42, −8.2)	<0.00001
Different research purpose:					
Intelligence quotient	[1], [2], [3], [11]	<0.00001	90.6	−12.21 (−18.47, −5.95)	<0.0001
Multifactor	[5], [6], [9]	0.004	81.8	−12.14 (−16.63, −7.61)	<0.00001
Conduct	[4], [8]	0.19	41.0	−18.36 (−23.81, −12.91)	<0.00001

### Supplementary subgroup analysis

**Figure 6:**
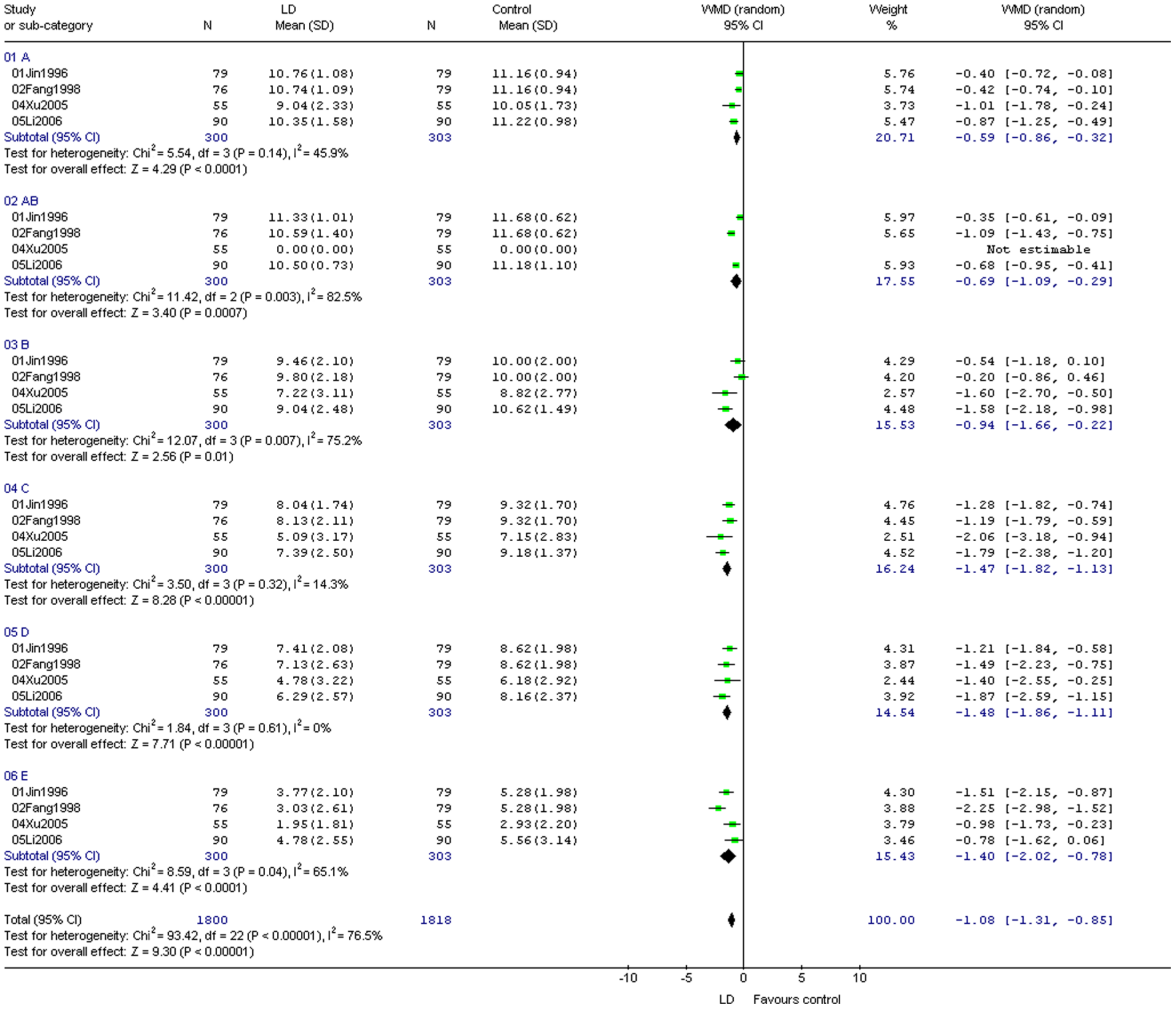
Meta-analysis of each sub item.

The results of the meta-analysis of each sub item showed that 5 studies had reported raw score points that had been measured [11]–[15]. The heterogenic sensitivity of the sum of the sub-measurement (I^2^) was 76.2%, (*P*<0.00001). In this way, the sub-measured items were heterogeneous, and **C** and **D** showed low heterogeneity, **A** showed moderate heterogeneity, and **AB**, **B**, and **E** were highly heterogeneous. The sub measurement was designed to range from easy to difficult. Throughout its value, regardless of group learning difficulties or normal comparative groups, scores showed a decreasing trend. For the first three sub-measurement items, the delay of children with learning difficulties was weaker (respective **WMD** values were −0.59, −0.69, and −0.94), all less than one test item. For the latter three sub-measurement items, the delay became 1–2 test items (**WMD** are −1.47, −1.48, and −1.40 respectively) (Figure 6).

## Discussion

In delayed fluid intelligence in children with learning difficulties, fluid analogy is the key to fluid cognition. In particular, it involves the blending of factors that adapt to new information to restructure and remodel creativity, from the clues of letters, numbers, and polygons. This reflects the fluid analogy and crystal analogy. The task reaction model of the frontal and temporal lobes toward the fluid intelligence were found to differ from that toward crystal intelligence [23]. Studies have confirmed that is more activity in the rear part of the brain (left **BA37/19**) of individuals who score more highly on the Raven Reasoning Test. This suggests that individual differences in the general intelligence category may be related to brain activity. Subjects with higher or lower general intelligence levels tend to favor different neural circuits, especially in non-frontal areas not related to information processing. The acquisition of primary factors of fluid intelligence may be important in learning difficulties for adults [24]. Children with learning difficulties were defined as children with otherwise normal intelligence who experienced delays or backsliding in the development of brain function. A syndrome of different degrees of difficulties in speaking and writing was observed. Meta-analysis of total IQ showed that total fluid intelligence of children with learning difficulties was more than 10 points behind that of normal children, supporting the hypothesis that despite the fact that most of the children with learning difficulties appeared to have normal intelligence, their development had been delayed.

There is good reason to use meta-analysis. The author has explored the relationship between the assessment of learning ability and Wechsler intelligence test results of children with learning difficulties. Analysis showed that the expressions and visual memory capacity of children with learning difficulties to be relatively low, and verbal expression all characteristics of auditory and visual perception to be positively associated with the space factor of Wechsler Intelligence [25]. Currently, there is a lack of in-depth research on fluid intelligence. The sub-measurement items for fluid intelligence tests were designed to include such matters as appearance perception and rational logical reasoning. The combined results of Chinese children with learning difficulties fell into two groups: **A-AB-B** and **C-D-E**. Within these groups, individuals showed similar delays, which does not exclude the effects of influencing factors. The **AB, B, E** group showed relatively high sensitivity. This could not have been observed in any single-item study. Intelligence testing is time-consuming, laborious, and requires a specific detection environment. Usually, the testing sample is small in scale, which results in a weak representation. Meta-analysis cam compensate for this striking deficiency.

There is also good reason to measure the fluid intelligence of children with learning difficulties. The biggest advantage of the Raven fluid intelligence test is it is not subject to social, cultural, or language influences. This gives allows results from different parts of the world to be compared easily. It also requires less time with respect to collective action and can be used as a pad for potential creative abilities. However, there are race, gender, and age differences among the participants. Weighting processes may be used to render these differences negligible. There are also hundreds of English articles that were not screened out. Many of these had design structures similar to the Chinese articles. This requires careful weighting. The activation, passing, and speed of brain nerves in different parts of the brain with respect to the recognition of Chinese characters may be slightly different from that involving the recognition of Western letters in individuals with learning difficulties [26]. For this reason, this paper only focuses on the weighted analysis of children with learning difficulties living in mainland China. The fluid and crystal intellectual measurements showed similar results, indicating that the children with learning difficulties had IQ scores more than 10 points lower the normal control group [27]. The Raven test was used, but its range was limited range exists to individual children with learning difficulties. The Wechsler test which focuses on and covers more of fluid crystal intelligence may better reflect the children current level of learning ability, particularity with respect to recognizing Chinese characters. In this way, it is more necessary evaluate of fluid and crystallized intelligence by weighted analysis and comparison, respectively, and to summarize representative findings. In any case, the delay in general fluid intelligence was not found to be sufficient to diagnose the crux. Other indicators must be used to supplement the evaluation of specific competencies.

## Conclusion

A meta-analysis of Raven fluid intelligence measurements of Chinese children with learning difficulties showed the overall IQ of the difficult group to be about 10 points lower than that of the control group. There was significant heterogeneity between studies. The delay in the sub-measure group **A**, **AB**, **B** was close, 0.4–0.9 points lower; the **C**, **D**, and **E** groups showed similar delays. They were 1.4–1.5 points apart. The high-sensitivity groups were **AB**, **B**, and **E**.

## Supporting Information

Checklist S1 **PRISMA checklist.**
(DOC)
