# Supplementary material for: Meta-Analysis of Fluid Intelligence Tests of Children from the Chinese Mainland with Learning Difficulties
Source: PLoS One. 2013 Nov 13;8(11):e78311. doi: 10.1371/journal.pone.0078311 (PMC3827259; doi:10.1371/journal.pone.0078311)
Supplement: Checklist S1 — PRISMA checklist. (DOC) [file pone.0078311.s001.doc]

| **Section/topic** | **#** | **Checklist item** |  |
| --- | --- | --- | --- |
| **TITLE** | | |  |
| Title | 1 | Identify the report as a meta-analysis. |  |
| **ABSTRACT** | | |  |
| Structured summary | 2 | Provide a structured summary including: objectives; data sources; study eligibility criteria, participants, and measurements; study appraisal and synthesis methods; results; limitations; conclusions. |  |
| **INTRODUCTION** | | |  |
| Rationale | 3 | Describe the rationale for the review in the context of what is already known. |  |
| Objectives | 4 | Provide an explicit statement of questions being addressed with reference to participants, measurement, comparisons, outcomes, and study design (PMCOS). |  |
| **METHODS** | | |  |
| Protocol and registration | 5 | Indicate the meta-analysis was a pre-established program with offline ReverMen. |  |
| Eligibility criteria | 6 | **Inclusion criteria:** Specify study characteristics as PMCOS, and study participants were school-age children (6–16 years old). They were of Chinese Han ethnicity. The study was comparative study, evaluating both normal children and children with learning difficulties. The general fluid intelligence tests were performed with the Combined Raven’s Test (CRT). Languages were limited to Chinese and English.​, All studies were performed in mainland China. Repeatedly published documents were included among recently published documents.  **Exclusion criteria:** The published article was written in dialect, not standard language. Either the control group contained non-normal children served as controls or comparisons were only made among groups of children with learning difficulties, even if those difficulties were of different levels of severity. A repeat publication. Brain functions delay were evaluated in the study.  **Diagnostic criteria and CRT basic contents** |  |
| Information sources | 7 | The information sources were from PubMed, MDConsult, the China National Publication Linker (cnpLINKer), the Chinese Knowledge Resource Integrated Database, and the Wan Fang Biomedical Journals Database. |  |
| Search | 8 | [http://www.mdconsult.com](http://www.mdconsult.com/); <http://www.yz365.com/Pubmed>; <http://192.168.106.13/kns50/>; [http://g.wanfangdata.com.cn](http://g.wanfangdata.com.cn/) |  |
| Study selection | 9 | Studies that met the inclusion criteria were accessed. |  |
| Data collection process | 10 | The first author, publication date, author’s organization, sample size, Chinese version of CRT, diagnostic criteria, form of publication, FIQ value, sub-item scores, and matching conditions and the subjects’ geographical location, age, and learning difficulties were recorded. |  |
| Data items | 11 | FIQ  List and define all variables for which data were sought (e.g., PICOS, funding sources) and any assumptions and simplifications made. |  |
| Risk of bias in individual studies | 12 | No. 11 study ( between genders), and No.12 study (among the LD types). |  |
| Summary measures | 13 | Difference in means |  |
| Synthesis of results | 14 | I^2^ |  |

Page 1 of 2

| **Section/topic** | **#** | **Checklist item** |  |
| --- | --- | --- | --- |
| Risk of bias across studies | 15 | publication bias, selective reporting within studies |  |
| Additional analyses | 16 | subgroup analyses |  |
| **RESULTS** | | |  |
| Study selection | 17 | The flow diagram (figure1). |  |
| Study characteristics | 18 | The study size, PMCOS (Table 1). |  |
| Risk of bias within studies | 19 | Data on risk of bias of each study (figure 2, 3). |  |
| Results of individual studies | 20 | A forest plot of FIQ (figure 4). |  |
| Synthesis of results | 21 | WMD of FIQ (figure 5) |  |
| Risk of bias across studies | 22 | The results of any assessment of risk of bias across studies (Table 2). |  |
| Additional analysis | 23 | subgroup analyses (figure 6) |  |
| **DISCUSSION** | | |  |
| Summary of evidence | 24 | Meta-analysis of total IQ showed that total fluid intelligence of children with learning difficulties was more than 10 points behind that of normal children, supporting the hypothesis that despite the fact that most of the children with learning difficulties appeared to have normal intelligence, their development had been delayed. The **AB, B, E** group showed relatively high sensitivity. |  |
| Limitations | 25 | Intelligence testing is time-consuming, laborious, and requires a specific detection environment. Usually, the testing sample is small in scale, which results in a weak representation. Meta-analysis can compensate for this striking deficiency. However, there are race, gender, and age differences among the participants. Weighting processes may be used to render these differences negligible. There are also hundreds of English articles that were not screened out. Many of these had design structures similar to the Chinese articles. This requires careful weighting. The activation, passing, and speed of brain nerves in different parts of the brain with respect to the recognition of Chinese characters may be slightly different from that involving the recognition of Western letters in individuals with learning difficulties |  |
| Conclusions | 26 | A meta-analysis of Raven fluid intelligence measurements of Chinese children with learning difficulties showed the overall IQ of the difficult group to be about 10 points lower than that of the control group. There was significant heterogeneity between studies. The delay in the sub-measure group **A**, **AB**, **B** was close, 0.4–0.9 points lower; the **C**, **D**, and **E** groups showed similar delays. They were 1.4 –1.5 points apart. The high-sensitivity groups were **AB**, **B**, and **E**. |  |
| **FUNDING** | | |  |
| Funding | 27 | There is no current funding, Professor Ting Zhang, the vise-president, will support the publication fee when the paper published with the Institute Foundation. |  |

*From:*  Moher D, Liberati A, Tetzlaff J, Altman DG, The PRISMA Group (2009). Preferred Reporting Items for Systematic Reviews and Meta-Analyses: The PRISMA Statement. PLoS Med 6(6): e1000097. doi:10.1371/journal.pmed1000097

For more information, visit: **www.prisma-statement.org**.

Page 2 of 2
